# Increasing cervical cancer screening in Iran: effectiveness of a theory-based educational intervention

**DOI:** 10.1186/s12978-022-01489-5

**Published:** 2022-09-01

**Authors:** Zahra Hosseini, Shokrollah Mohseni, Rahimeh Momeni, Teamur Aghamolaei, Azin Alavi, Sara Dadipoor

**Affiliations:** 1 Social Determinants in Health Promotion Research Center, Hormozgan Health Institute, Hormozgan University of Medical Sciences, Bandar Abbas, Iran; 2 Student Research Committee, Hormozgan University of Medical Sciences, Bandar Abbas, Iran; 3 Cardiovascular Research Center, Hormozgan University of Medical Sciences, Bandar Abbas, Iran; 4 Mother and Child Welfare Research Center, Hormozgan University of Medical Sciences, Bandar Abbas, Iran

**Keywords:** Cervical cancer, Screening behavior, Women, BASNEF model

## Abstract

**Purpose of study:**

The high mortality rate of cervical cancer in developing countries is mainly related to inefficient screening programs. The aim of the present study was, thus, to determine the effect of an educational intervention based on BASNEF (Belief, Attitudes, Subjective Norms, and Enabling Factors) model on increasing the rate of cervical cancer screening (CCS) in Bandar Deir in the south of Iran.

**Methods:**

A quasi-experimental educational intervention was made with 202 women participants (101 in the intervention group (IG) and 101 in the control group (CG)) in 2019–20. The sampling was convenience in type. The data were collected using a reliable and valid tripartite questionnaire (demographic information, knowledge, BASNEF constructs). A total number of 14 training sessions were held each taking 60 min, at two levels, personal and interpersonal (for family members, health workers and healthcare givers). Finally, there was a three-month follow-up held in December 2021.

**Results:**

After the training, a statistically significant difference was found between the IG and CG in all model constructs (p < 0.001). Before the intervention, in the IG, the personal health score was 4.35 ± 2.52, which was increased to 5.25 ± 0.753 after the training (p < 0.001). However, in the CG, the difference was not statistically significant (p < 0.030). 63.4% of women in the IG and 32.7% in the CG performed the CCS and the between-group difference was statistically significant (p < 0.001). Attitude, enabling factors and behavioral intention were the main predictors of CCS.

**Conclusion:**

The present findings showed though the training intervention based on the BASNEF model had limited resources and was run in a short time, it managed to motivate women to perform the CCS. It could maximally remove barriers at both personal and interpersonal levels and suggest strategies in the light of these barriers to achieve a successful screening program.

**Supplementary Information:**

The online version contains supplementary material available at 10.1186/s12978-022-01489-5.

## Plain language summary

Cervical cancer is the fourth most prevalent fatal cancer among women on a global scale, and is one of the few cancers that can be prevented through screening. The high mortality rate of cervical cancer in developing countries, including Iran, has been largely attributed to inefficient screening programs. In developing countries cervical cancer screening programs are far from qualified. Yet, theory-based educational interventions can play an important role in promoting CCS behavior. Therefore, the present study was designed to determine the effect of an educational intervention based on the Beliefs, Attitudes, Subjective Norms and Enabling Factors (BASNEF model on increasing CCS in women in the south of Iran. BASNEF model includes beliefs about behavioral outcomes, attitudes toward behavior, subjective norms and enabling factors. The most significant construct in BASNEF model is the behavior change. Beliefs and attitudes are influenced by culture, values, traditions, education, media, and personal experiences. Subjective norms include family, society, social media, and peer pressure. Enabling factors include income, women’s status, time, and skills. This model can be proposed as a new educational method. A quasi-experimental two-group (pretest–posttest) study was conducted in 2019–2020 on 202 women (101 in the intervention group, and 101 in the control group). In the intervention group, 14 training sessions (10 at the personal level and 4 at the interpersonal level) were held between 40 and 60 min’ long. In the control group, 1 training session was held in 6 groups of 15–20. It was 60 min’ long. The follow-up was held three months after the intervention. The results showed that the educational intervention, by affecting the BASNEF constructs, significantly affected women’s CCS behavior. Maximizing barriers at both personal and interpersonal levels and suggesting strategies based on these barriers can help achieve a successful screening program. In particular, we call for the implementation of goal-oriented training programs according to health education and health promotion models to increase the rate of CCS behavior.

**Supplementary Information:**

The online version contains supplementary material available at 10.1186/s12978-022-01489-5.

## Introduction

Cervical cancer is the fourth most prevalent and fatal cancer among women worldwide [1]. Global Cancer Statistics (2020) showed 604,127 women were diagnosed with cervical cancer on a global scale, and about 341,831women died from the disease. About 90% of the new cases and deaths induced by cervical cancer worldwide in 2020 occurred in low- and middle-income countries [2].

The incidence rate of cervical cancer in Iran is on the rise [3]. In Iran, the incidence rate of the disease is 4.5 per 100,000 people. Annually, among every 123 women, one is affected with cervical cancer, and among every 100,000 women, nine die of this cancer [4]. The most important risk factors of the disease are the human papillomavirus (HPV) infection (approximately accounting for 80–99% of all cervical cancer) being HIV positive [5, 6], pregnancy at young age, young age at marriage, multiple sexual partners, use of handmade sanitary napkins, failure to wash genitals after sexual intercourse, low immune system, genetic factors, and exposure to certain chemicals [7].

Women with cervical cancer suffer from different clinical symptoms including social malfunctioning, constipation, diarrhea, severe lymphedema, menopausal symptoms, reduced body image, sexual or vaginal functioning, anxiety and depression, and difficulty managing finance [8, 9].

Invasive cervical cancer has been identified as a preventable cancer due to its long pre-invasive cycle, the availability of an appropriate screening program, and the effective treatment of primary lesions [10]. The occurrence of this cancer can be prevented by regular screening services [11]. The high cervical cancer mortality in developing countries is mainly due to inefficient screening programs, limited access to cervical cancer screening, and low-quality treatments after abnormal outcomes [12]. Prompt and accurate screening programs are essential for every woman with a cervical disease to receive the treatment she needs and to escape an avoidable death [13].

The performance rate of CCS programs in Iran is below the optimal level, and significantly needs improvement. As for the distribution of CCS behavior, social and geographical differences show the need for further research and more comprehensive strategies to reduce the rate of the disease and increase the performance rate [14].

A main barrier to CCS is the women’s inadequate knowledge of the disease, the essentiality of screening, and unfamiliarity with the screening sites [11]. Iranian women’s knowledge of, attitude toward and performance of CCS are far from satisfactory [15, 16]. In fact, educational interventions can play an important role in promoting CCS behavior in women [17–19]. Theory-based educational interventions have a better chance of success than simple educational interventions. The most effective educational programs are based on theory-based approaches that stem from the behavior change models.[20]. The existing body of research proved the effectiveness of theory-based educational interventions in increasing the rate of performing CCS [12, 21, 22]. A systematic review report showed that various interventions aiming to change health behaviors were successful in preventing cervical cancer [17]. A successful educational model of behavior change or creation of a new behavior is the BASNEF model. Different studies confirmed the effectiveness of educational interventions based on this model in promoting healthy behaviors. [23, 24]. One advantage of BASNEF model is that, unlike other models, it not only considers knowledge, attitude, abstract norms, and intention, but also focuses on another influential factor, namely the enabling factors. As a catalyst, intention turns into actual behavior, and its absence disrupts the process. A relevant study reported the barriers including enabling factors among the reasons for women’s low adherence to the CCS [25]. Other researches considered the lack of enabling factors, including the ability to access and pay for health services, among the barriers to cervical cancer screening [26, 27].

BASNEF model, as described by John Hubley, includes beliefs about behavioral outcomes, attitudes toward behavior, subjective norms and enabling factors. This model is a combination of the Precede–Proceed model and the expectancy-value theory [28, 29]. The most significant construct in BASNEF model is the behavior change. Beliefs and attitudes are influenced by culture, values, traditions, education, media, and personal experiences. Subjective norms include family, society, social media, and peer pressure. Enabling factors can be income, women’s status, time, and skills [29].

**Fig. 1:**
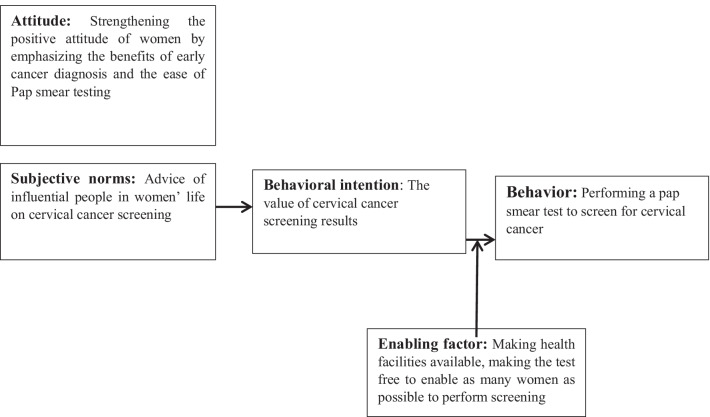
The conceptual framework of this research

According to this model, an individual performs CCS only when she perceives the benefits and importance of CCS and develops a positive attitude towards this behavior. In addition, she needs to be encouraged by influential people in her life to perform CCS. Resources and facilities should be taken into account too (Fig. 1).

Most previous studies explored the increase in CCS with models such as the Health Belief Model and Theory of Planning Behavior [22, 30, 31]. To our knowledge based on the literature review, only one study used BASNEF model in increasing CCS. The study site, sample size, type of educational intervention and its statistical analysis were, however, different from our study [32]. This study has explored the effect of an educational intervention based on BASNEF model on increasing CCS. The results of this research provide researchers with primary insights into the effectiveness of this model and a basis for comparison with future research using this model.

## Methods

### Study design and population

The present quasi-experimental study was conducted with an experiment group. It was carried out in 2019–2020 to assess the effectiveness of an educational intervention based on the BASNEF model in promoting CCS behavior among 202 women between 20 and 49 years of age (101 in the IG and 101 in the CG). It was conducted in the south of Iran.

### Setting

The present study was conducted in Deir County in Bushehr Province in the south of Iran. This place is located on the coastal areas of the Persian Gulf, and the capital city is Bandar-Deir.

### Eligibility criteria

The inclusion criteria were: 20–49 years of age, being sexually active, not being pregnant, being literate (i.e., being able to read and write, to jot down their ideas themselves to reduce any bias) and full consent to participate in the training sessions. The exclusion criteria were: absence at more than two training sessions, absence at the post-test, history of uterine cancer, hysterectomy and other sexually transmitted diseases such as Chlamydia infections, Neisseria gonorrhoeae, Herpes simplex virus, Trichomonas vaginalis diagnosed by a gynecologist assistant in this project, unwillingness to continue participation in the study, and incomplete questionnaires.

### Sample size estimation

The sample size was estimated using the following formula:

$$n = \frac{{2(z_{1 - \alpha /2} + z_{1 - \beta } )^{2} \sigma^{2} }}{{d^{2} }} = \frac{{2*(1.96 + 0.84)^{2} *(12)^{2} }}{{5^{2} }} = 91$$

The sample size in each group was calculated as 91. With an attrition rate of 10% in each group, the final sample size was decided to be 101 (for each group).

### Sampling

There are only two comprehensive healthcare centers in Deir County. In order to prevent the information exchange between the intervention and control groups, one of these centers was randomly selected as the intervention group and the other as the control. Thus, healthcare center #1 was selected as the IG and #2 as the CG. Then, women who met the inclusion criteria were assigned to each group.

### Content of the survey instrument and scoring system

The questionnaire used in this study contained closed-ended questions to be rated on a Likert scale, true, false, or do not know. This questionnaire was divided into three main parts.

The first part contained questions exploring the participants’ demographic information including age, education, husband’s education, residence, occupation, history of the Pap test.

The second part consisted of questions about knowledge of causes of the disease, risk factors, prevention and therapeutic measures. This part included 15 three-choice questions. The choices were True, False, and do not know. Every true answer received 1, and a false or don’t know received 0.

The third part included the BASNEF constructs as summarized in Table 1.

**Table 1:** Description of the research instrument

Constructs	No. of items (scale)	Scoring (range)	Internal consistency (Cronbach's alpha)	Sample item
Knowledge	15 items (Multiple Choice Questions)	True/ False/ Don't know	0.88	Abnormal bleeding is a symptom of cervical cancer
Attitude	10 items (Likert Scale Questions)	Strongly Agree = 1, Agree = 2, neutral 3, Disagree = 4 Strongly Disagree = 5	0.86	Performing the pap test is easier than treating cervical cancer
Subjective norms	16 items Likert Scale Questions)	Strongly Agree = 1, Agree = 2, neutral 3, Disagree = 4 Strongly Disagree = 5	0.78	My husband does not consent to paying for the Pap test
Enabling factor	6 items (Likert Scale Questions)	Strongly Agree = 1, Agree = 2, neutral 3, Disagree = 4 Strongly Disagree = 50	0.86	If the test is free of charge, I will be able to do the screening
Behavioral intention	5items (Likert Scale Questions)	Strongly Agree = 1, Agree = 2, neutral 3, Disagree = 4 Strongly Disagree = 5	0.8	I am planning to regularly have the pap test (once a year for up to 3 years if there are no problems, once in 3 years)
Personal health	6 Item (Dichotomous Question)	Yes/No	0.84	I do the pap test regularly (once a year for up to 3 years if there are no problems, one in 3 years)

All items of the subscales were rated on a five-point Likert-scale: strongly agree (1 point), agree (2 points), undecided (3 points), disagree (4 points), and strongly disagree (5 points). Each subscale was assessed separately, and the total score was not calculated. Subscale scores were calculated for each participant. Higher scores indicated stronger feelings for a construct.

### Data quality assurance

The data collection instrument was developed based on a review of the related literature. The questions were pretested and well-organized. All were to be rated as self-reports. Before the main data collection phase, the questionnaire was piloted on 23 women who were similar to the research population on all aspects. Their feedback was used to revise the content and facilitate its comprehension and organization. These participants were excluded from the main phase of study. Also, the initial draft of the questionnaire was sent to a panel of experts to evaluate the readability, simplicity, relevance, and importance criteria. Their opinions were used to improve the organization and content of the questionnaire. To determine the reliability of the instrument, a test–retest method was used. The questionnaire was given to 20 individuals who had the same conditions as the subjects at a two-week interval and on two occasions. Then, to calculate the test agreement with the retest, the ICC index was estimated. The estimated ICC value was 0.86; thus, the reliability of the questionnaire was substantiated.

### Data collection

The data were collected using self-administered questionnaires. A written consent form was completed in two copies. One remained with the researcher and the other with the participant. After obtaining the written consent, complete explanations were provided to the two groups (control and intervention) about the procedure. Then the pre-test questionnaires were submitted to the two groups. Three months after the training, to assess the effectiveness of the educational intervention, the two research groups completed the post-test questionnaires again.

At the interpersonal level, the research participants included the women’s family members and health care workers accessed by the corresponding author through a snowball sampling. The purpose of the training at this level was to strengthen the social skills (the effect of women’s friends on increasing their motivation to perform the screening test). Therefore, no questionnaire was distributed among these participants before and after the training.

Of note is that the questionnaires were distributed by the same member of the research team (the corresponding author) in the morning shift in the training room of each health center. In order to tell apart the pre-test and post-test questionnaires, each participant entered the last 4 digits of the mobile phone number and their age on the questionnaire. Each questionnaire took 20–25 min to complete.

### Intervention activities and follow-up

In the present study, the educational intervention was carried out at both personal and interpersonal levels. For the former, the training was held for the women. The relevant questionnaires (awareness and constructs of BAZNEF model) were used as the pre-test for the women of the IG. Then according to the results obtained from the pre-test, an educational needs assessment was done to decide on the educational materials and methods and the number of sessions needed. The teaching methods included lectures, collaborative discussions, Q&As, brainstorming, role models, peer education. The educational content of each session was tailored to the comprehension level of the learners, use of reliable scientific sources, inclusion of experts’ as well as participants’ opinions, and the Baznef model constructs. A total number of 10 training sessions were held in 6 groups, each session lasting for 40–60 min, and a 10-min break included.

At the interpersonal level, the training was held for family members, patients and health care workers. At this level, 4 training sessions were held for 6 groups, each session taking 40–60 min with a 10-min break.

It should be noted that in order to adjust the enabling factor, the sampling and testing were made free of charge for the participants of the IG. The cost of the above tests was paid from the budget provided by the supporting organization.

In the CG, pre-test questionnaires were completed at the same time as the intervention group. Then, a one-hour training session was held on the significance of the CCS in 6 training groups of 15–20 members. Three months after the training, the post-test questionnaires were distributed among the participants. The CG did not attend the training specifically held for the IG (based on the BAZNEF constructs, making the pap test free to adjust the enabling factors, etc.). For example, making the test free (for the effect of the enabling factor, which is one of the components of the BEZENF model) was implemented only in the IG. The reason was to assess the real effect of the educational intervention on the screening behavior of cervical cancer in women.

It is noteworthy that all educational interventions were designed by the research team and implemented in the training room of the relevant health center.

**Fig. 2:**
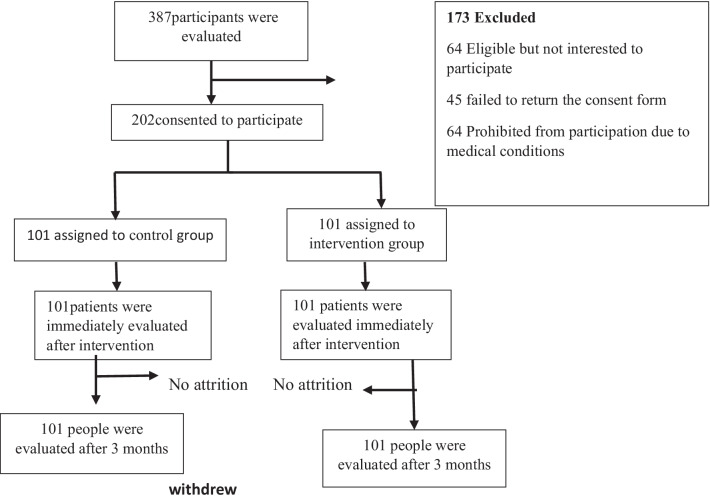
Intervention profile

In order to ensure minimal contamination of groups, the women of the IG and CG were selected from two comprehensive health service centers that were far from each other, so that the chances of exchanging information between the two groups were minimized. Also, during the intervention program, no training was held by other organizations. Nor was any other relevant public training held in the whole city. It was attempted to reduce the attrition rate using telephone follow-ups once a week. Thus, after three months, there was no attrition in the intervention and control groups (Fig. 2). Details of the educational intervention are provided in Additional file 1.

### Post intervention activities

Three months after the training, the questionnaires were completed by both groups to assess the effectiveness of the educational intervention.

The educational intervention covered the following topics.

1. General issues with cancers and more specifically the cervical cancer, detailed description of cervical cancer (how people can prevent the risk of cervical cancer, etc.), factors affecting cervical cancer
2. Symptoms of the disease, prevention and therapeutic measures
3. Benefits of early diagnosis of cervical cancer, introduction of cervical cancer screening test
4. Teaching the benefits of CCS, time of cervical cancer screening, place of cervical cancer screening.

### Interventionist training

The educational intervention was instructed by (1) SD (a health promotion/education researcher experienced in the field of educational interventions based on models of health education and health promotion), (2) A gynecologist with more than 15 years of educational experience (3) A group of peers.

### Output evaluation

Knowledge, Attitude, Subjective norms, Enabling factors, Behavioral intention.

### Outcome evaluation

Cervical cancer screening (whether or not a woman has the Pap test).

### Ethical considerations

For data collection, we visited the comprehensive healthcare centers with an official introduction letter from the deputy of research. First, we introduced the research objectives in full and in a simple and clear manner. All details of the research were explained and the participants were asked for a voluntary participation. They were then asked not to mention their names. They were also assured of the confidentiality of the information they provided. The research project was approved by the ethics committee of Hormozgan University of medical sciences (#IR.HUMS.REC.1398.112).

### Data management and analyses

When the data collection was over, descriptive statistics (frequency, relative frequency, mean and standard deviation) were used to describe participants’ age, education, husband’s education, and occupation. Kolmogorov–Smirnov test and Levene’s test were run to check the normality of distribution and equality of variances. For inferential statistics, the independent-sample *T* test was run to compare model constructs and the performance of CCS in the IG and CG. Paired-samples *T*-Test was run to compare the scores of the model constructs before and after the educational intervention within each group. ANCOVA was used to control and adjust for the effect of pre-intervention scores on post-intervention scores. Also, to evaluate the effect of each model construct on behavior in the IG, multiple linear regression analysis was used, in which the behavioral constructs were considered as dependent variables and knowledge and the model constructs as the independent variables. All analyses were done in SPSS 22.

## Results

### Participants’ descriptors

The mean and standard deviation of participants’ age in the IG and CG were, respectively, 32.90 ± 7.27 and 32.59 ± 6.36 years. The highest frequency of education level in both groups belonged to high school education (44.6% in the IG and 34.7% in the CG). In both groups, the majority of participants were housewives (94.1% in the IG and 82.2% in CG). 27.7% of the CG and 41.6% of the IG had a history of CCS. Other demographic information is summarized in Table 2.

**Table 2:** Comparison of demographic variables between the research groups in 2019–2020, Iran

Category	IG		CG		P-value*
	Number	Percentage (%)	Number	Percentage (%)	
Age (M, SD)	32.90 ± 7.27	–	32.59 ± 6.36	–	0.75
Education					0.324
Elementary	21	20.8%	19	18.8%	
Intermediate	21	20.8%	25	24.8%	
High school	45	44.6%	35	34.7%	
Collegiate	14	13.9%	22	21.8%	
Husband's education					0.95
Primary	19	18.8%	21	20.8%	
Secondary	29	28.7%	31	30.7%	
Diploma	24	23.8%	23	22.8%	
Academic	29	28.7%	26	25.7%	
Residence					1
Urban	70	69.3%	70	69.3%	
Rural	31	30.7%	31	30.7%	
Occupation					0.009
Housewife	95	94.1%	83	82.2%	
Working outside home	6	5.9%	18	17.8%	
Previous screening					0.631
Yes	28	27.7%	25	24.7%	
No	73	72.3%	76	75.3%	

*Chi-square test

### Findings for BASNEF constructs

Before the intervention, the two groups differed significantly in terms of the score of Enabling factors (p = 0.024). However, the two groups did not differ significantly in other constructs (of BASNEF model) (P > 0.05). After the educational intervention, a statistically significant difference was found between the IG and CG in all constructs (p < 0.001). In the IG, before the educational intervention, the personal health score was 4.35 ± 5.25, which was increased to 5.25 ± 0.753 after the intervention (p < 0.001). In the CG, this difference was not statistically significant (p < 0.030) (Table 3).

**Table 3:** Comparison of the BASNEF constructs between the two research groups before and after the intervention 2019–2020, Iran

Variable	GroupTime	IG Mean ± SD	CG Mean ± SD	P-value*
Knowledge	Baseline	5.36 ± 3.34	6.47 ± 3.15	0.006
	3-months follow-up	10.95 ± 1.54	6.70 ± 3.09	0
	P-value**	0.001	0.206	
Attitude	Baseline	28.26 ± 6.18	27.55 ± 5.51	0.531
	3-months follow-up	34.19 ± 5.29	27.14 ± 5.09	0
	P-value**	0	0.17	
Subjective norms	Baseline	42.88 ± 9.87	41.98 ± 8.74	0.556
	3-months follow-up	50.42 ± 6.45	42.54 ± 8.27	0
	P-value**	0	0.276	
Enabling factor	Baseline	13.30 ± 5.60	14.91 ± 5.49	0.024
	3-months follow-up	22.22 ± 3.11	14.81 ± 4.81	0
	P-value**	0.002	0.637	
Behavioral intention	Baseline	14.16 ± 3.71	13.89 ± 3.00	0.516
	3-months follow-up	17.64 ± 2.21	13.81 ± 2.95	0
	P-value**	0	0.615	
Personal health	Baseline	4.35 ± 5.25	4.31 ± 1.18	0.808
	3-months follow-up	5.25 ± .753	4.46 ± .92	0
	P-value**	0.000	0.030	

*Independent T-test; **Paired T-test

To control and adjust for the effects of pre-intervention scores, ANCOVA was run. As reported in Table 4, pre-intervention scores as a covariate of knowledge constructs (partial η^2^ = 0.394; p < 0.001), attitude (partial η^2^ = 0.486; p < 0.001), social norms (partial η^2^ = 0.512; p < 0.001), enabling factors (partial η^2^ = 0.442; p < 0.001), and intention (partial η^2^ = 0.442; p < 0.001) were significantly effective. It can also be seen, in the same table, that the educational intervention has a significant effect on all model constructs including knowledge (partial η^2^ = 0.606; p < 0.001), attitude (partial η^2^ = 0.446; p < 0.001), social norms (partial η^2^ = 0.338; p < 0.001), enabling factors (partial η^2^ = 0.644; p < 0.001), and behavioral intention (partial η^2^ = 0.474; p < 0.001).

**Table4:** Covariance analysis of BASNEF constructs 2019–2020, Iran

Variables	Source	Sum of squares	df	Mean square	Statistic F	p-value	Partial Eta squared
Knowledge	Before intervention	445.398	1	445.398	122.015	0.000	0.394
	Intervention	1057.158	1	1057.158	289.604	0.000	0.606
	Error	686.267	188	3.650			
	R Squared = 656 (Adjusted R Squared = 652)						
Attitude	Before intervention	2480.820	1	2480.820	179.879	0.000	0.486
	Intervention	2107.335	1	2107.335	152.799	0.000	0.446
	Error	2620.399	190	13.792			
	R Squared = 0.651 (Adjusted R Squared = 0.647)						
Subjective norms	Before intervention	5322.463	1	5322.463	197.573	0.000	0.512
	Intervention	2587.758	1	2587.758	96.059	0.000	0.338
	Error	5064.563	188	26.939			
	R Squared = 0.621 (Adjusted R Squared = 0.617)						
Enabling factor	Before intervention	1390.170	1	1390.170	150.636	0.000	0.442
	Intervention	3175.929	1	3175.929	344.137	0.000	0.644
	Error	1753.448	190	9.229			
	R Squared = 0.697 (Adjusted R Squared = 0.694)						
Behavioral intention	Before intervention	576.167	1	576.167	150.280	0.000	0.442
	Intervention	656.873	1	656.873	171.330	0.000	0.474
	Error	728.452	190	3.834			
	R Squared = 0.638 (Adjusted R Squared = 0.634)						

Multivariate linear regression analysis was used to evaluate the effect of each model construct on behavior. Behavior was the dependent variable, while knowledge and other constructs were the independent variables. As shown in Table 5, attitude, enabling factors and behavioral intention were the predictors of the desired behavior. Adjusted R2 = 0.479 indicates that this model managed to explain 47.9% of the behavioral score changes in the IG.

**Table 5:** Multivariate regression analysis of the predictors of behavior in the intervention group based on the model constructs 2019–2020, Iran

Variables	B	95.0% Confidence interval for B		Standardized coefficients Beta	T	p-value
		Lower bound	Upper bound			
Knowledge	− 0.060	− 0.144	0.024	− 0.123	− 1.423	0.158
Attitude	0.040	0.011	0.069	0.281	2.757	0.007
Subjective norms	0.004	− 0.015	0.024	0.038	0.453	0.652
Enabling factor	0.196	0.138	0.253	0.625	6.743	< 0.001
Behavioral intention	0.167	0.099	0.235	0.490	4.868	< 0.001
R Square = 0.507 Adjusted R Square = 0.479						

**Fig. 3:**
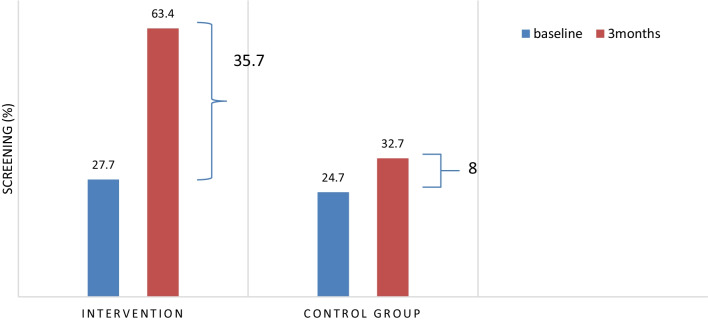
Comparison of CCS between the two groups before and after intervention in 2019–2020, Iran

Comparing CCS between the two groups before and after the intervention is summarized in Fig. 3

## Discussion

The present study evaluated the effect of an educational intervention developed based on the BASNEF model on promoting CCS behavior. The multivariate regression model (R^2^ = 0.47) showed that the independent variables integrated within the model (knowledge and BAZNEF constructs) explained 47% of the variance of the dependent variable (performing CCS).

The present findings showed that the designed educational program managed to significantly increase knowledge, BASNEF model constructs and the CCS behavior in women in the IG. Based on multivariate linear regression findings, attitude, enabling factors and behavioral intention were significant predictors of behavior.

After the training, a significant difference was found in the mean knowledge score of the IG compared to the CG. Similarly, in other educational interventional studies, the training showed to significantly affect the mean knowledge score of the IG compared to the CG [4, 32, 33]. In the present study, women’s knowledge in the IG became twofold, which indicates the success of the training in removing some misconceptions about the causes, risk symptoms and alternatives for preventing and treating cervical cancer. It should be noted that although the mean knowledge score in the IG was increased, this construct was not able to predict CCS behavior. In other words, women did not perform the CCS despite the knowledge. Similarly, in another study, awareness-raising did not manage to predict a higher CCS behavior [34]. Arguably, although awareness-raising is not directly related to the CCS behavior, it indirectly affects the increasing rate of CCS by affecting some other variables. Knowledge is an important factor in the success of disease prevention programs. Familiarizing participants with the cause of a disease along with an early diagnosis of the disease can be an important step in changing patients’ behavior. In this regard, a study in Iran showed that, by promoting knowledge of the causes of cervical cancer and its potential consequences, Iranian women can be encouraged to show behaviors that prevent cervical cancer [35].

The present study found an increase in the mean attitude score of IG compared to the CG. Another study also confirmed this finding, in which the educational intervention affected women’s attitude toward CCS [4, 22, 32]. Unlike the present study, in another research, the educational intervention did not manage to change women’s attitude [36]. Demographic features, duration and type of the training can be among the possible reasons for this discrepancy. In our study, attitude was one predictor of CCS behavior. It points to the fact that a theory-based and targeted educational intervention managed to raise awareness (increase women’s susceptibility to and understanding of the potential consequences of cervical cancer). Controlling barriers such as the cost of screening and knowing the reliable screening sites can positively affect women’s attitude. A qualitative study showed that creating a positive attitude towards screening behavior may encourage Iranian women to engage in cervical cancer prevention behaviors [35].

The mean subjective norms of the IG increased significantly compared to the control group. Similarly, in other studies, educational interventions managed to affect subjective norms [22, 32]. Although our educational intervention affected subjective norms, this construct did not predict the CCS behavior. Contrary to our research, in another study, subjective norms showed to predict the CCS behavior [37]. One possible reason for this discrepancy could be the different study designs. The present research is interventional while Moradi’s is cross-sectional. Of note is that the participants selected for training (among women’s acquaintances) were not close enough to the women so as to adequately affect their behavior. It is suggested in future research to ask each participant to nominate an influential person in life so that the educational intervention can prove effective [38].

In the present research, the mean score of enabling factors in the IG was significantly increased compared to the CG. Similarly, another study showed that the educational intervention was able to increase the mean enabling factor score for CCS [32]. In some research, women who were more aware of the screening sites and those who were screened for free were more likely to seek screening services [12]. In addition, enabling factors could predict screening behaviors in women, which was somewhat predictable because, in our study, free CCS and raising women’s awareness of screening sites and the like were used to control the enabling factors.

As the present findings showed, the IG intended more to show CCS behaviors than the CG. The between-group difference was statistically significant. Similarly, a body of research showed the success of educational interventions in increasing the intention to perform CCS [12, 32, 39]. Contrary to our findings, in another study, educational intervention had no effect on the intention of CCS [22]. The difference in the type of theory used can be one reason for divergent findings. In our study, the enabling factors, as a construct of the BASNEF model, were used as a catalyst between intention and actual behavior. It can help to change intention to behavior. It is noteworthy that not all women who intended to perform the screening did it actually. Behavior has been considered by researchers in the present study. In future research, if all barriers are taken into account, based on those barriers, resources and facilities (enabling factors) will be moderated. We can significantly turn the intention into a successful behavior.

Our findings showed that the CCS rate in the IG was increased significantly compared to the CG. Women in the IG underwent the Pap test almost twice as frequently as women in the CG. In agreement with this finding, some other studies showed that educational intervention can improve CCS behavior [4, 32]. Contrary to our finding, in another study, educational intervention had no effect on CCS behavior [22, 33]. The different socio-demographic features of research populations, educational content and types of model used are among reasons for this discrepancy. The success of the present study in improving attitude to CCS can be attributed to several factors. First, the population had very little previous knowledge of CCS in preventing cervical cancer, which increased significantly after the intervention. Freeing up the screening program was one enabling factor and theoretical and purposeful training through peers, gynecologist and experienced health education specialist were among other reasons that can be brought. In addition to success in promoting the acceptance of CCS, it is noteworthy that a number of women in the IG did not perform the screening despite the training. Arguably, the participants’ demographic characteristics may have influenced whether or not they intended to go for the screening. For instance, one study reported misconceptions about old age and menopause as the potential reasons for reducing women's susceptibility to cervical cancer. It could adversely affect the participants’ screening behavior [40].

### Limitations and strengths

There are several limitations to raise here. One is that the present study was quasi-experimental in design and used a convenience sampling method. Because participants were not randomly assigned to the IG and CG, interpreting the results should be done with caution. The pre-test, post-test and selection of the matched control group partially made up for this limitation. This research was conducted in a southern province of the country, which may not represent the total population of Iran, but the results can, with caution, be generalized to the southern cities of Iran with similar cultural contexts. The contamination between the two groups was another potential limitation of this study. Possibly, participants in the IG had access to the intervention information through acquaintances and the participants of the IG. However, the statistically significant between-group differences largely removed this bias. Not including illiterate women was another limitation of the present research. The strengths of this study are the inclusion of a matched control group, interpersonal educational intervention (intervention made by important and influential people, empowering resources), and inclusion of a three-month follow-up. The present study, in a short time with minimal facilities, managed to provide important information to policy makers in adopting cervical cancer preventive behaviors. These results could potentially be used in similar settings to increase the rate of low-resource CCS.

### Recommendations for further research

In our study, though a number of women in the IG received the same type of training in similar circumstances, they did not perform a Pap test as expected. Probably, the barriers to successful CCS may be beyond participants’ and researchers’ control. In another study, from a wide list of reasons for not screening for cervical cancer, the majority of women selected the other option. It shows that the options listed were not comprehensive enough and the barriers were more than those already anticipated and enlisted [11]. It seems that more comprehensive and multi-level trainings can better change women’s behavior. Thus, it is recommended to assess a research population’s educational and cultural needs before any interventional measures, because behavioral and environmental factors might impede women from performing the CCS. These factors need to be identified to guide the design of systematic and effective educational interventions at different levels (personal and interpersonal). Qualitative research can help further identify barriers to screening in the target population so as to overcome them. It is also suggested that future research use ecological models that take into account environmental factors in addition to individual factors to further increase the rate of CCS.

## Conclusion

The present findings showed that a low-cost educational intervention can promote CCS behavior. The educational intervention positively influenced women's health behavior by affecting the BASNEF model. Maximizing barriers at both personal and interpersonal levels and providing strategies based on these barriers can help to achieve a successful screening program. In particular, we call for the implementation of targeted training programs within the framework of health education and health promotion models to increase the rate of CCS.

## Supplementary Information

**Additional file 1.** Education and training content.

## Data Availability

The datasets used and/or analyzed during the study are available from the corresponding author on reasonable request**.**

## Abbreviations

CG: Control group
IG: Intervention group
CCS: Cervical cancer screening
CC: Cervical cancer
