# Supplementary material for: Increasing cervical cancer screening in Iran: effectiveness of a theory-based educational intervention
Source: Reprod Health. 2022 Sep 1;19:186. doi: 10.1186/s12978-022-01489-5 (PMC9438284; doi:10.1186/s12978-022-01489-5)
Supplement: Supplementary file 1 — Additional file 1. Education and training content. [file 12978_2022_1489_MOESM1_ESM.docx]

**Supplementary file 1. Education and training content**

| **Personal level** | | |
| --- | --- | --- |
| **Timing** | **Title** | **Activities** |
| 1^st^ week/60 minutes’ long | Getting to know the goals of the educational intervention, expectations and the process of conducting the educational intervention, gaining the trust of the participants | In this session, the trainer introduces the participants with the objectives and significance of study, informs them about the training methods and the educational content. A checklist which has been designed beforehand, is given to participants to rate the abovementioned qualities. Their feedback will be used to revise the content and procedures involved. |
| 2^nd^ week/60 minutes’ long | General information about cancers with a focus on cervical cancer, detailed description of cervical cancer | In this session, the women receive the training on cervical cancer through lectures, brainstorming, active collaborative discussion and viewing images on OHP as well as movies. At the end of the session, the evaluation checklist is completed to detect the deficiencies and fix them. |
| 3^rd^ week/60 minutes’ long | Getting to know the risk factors of cervical cancer | In this session, the women receive training (by the trainer) on the risk factors of cervical cancer using lectures, active participatory discussion and brainstorming. At the end of the session, the evaluation checklist is completed to detect the deficiencies and revise them. |
| 4^th^ week/40 minutes’ long | Getting to know the symptoms of cervical cancer | In this session, women are introduced to the symptoms of cervical cancer by the trainer through lectures, active collaborative discussion and brainstorming. |
| 5^th^ week/40 minutes’ long | Getting to know how to prevent cervical cancer | In this session, the women are introduced to the ways of preventing cervical cancer, the importance of testing and the places of testing. The instructions were provided through lectures, active participatory discussion, brainstorming, peer education by the trainer and 2 women who regularly performed cervical cancer prevention behaviors and were introduced to us through health care providers. |
| 6^th^ week/40 minutes’ long | Continue: Getting to know how to prevent cervical cancer | In this session, women are introduced to the ways of preventing uterine cancer, the importance of testing, and the places of testing by the trainer through lectures, active collaborative discussions, brainstorming, and peer education. |
| 7^th^ week/60 minutes’ long | Introduction of cervical cancer screening test, importance of cervical cancer screening, benefits of early diagnosis of cervical cancer, how to perform the screening, teaching suitable positions to reduce pain during sampling. | In this session, women are introduced to the benefits of early diagnosis of cervical cancer and appropriate positions to reduce pain during sampling. This information was provided by the trainer through lectures, peer education and practical demonstrations. |
| 8^th^ week/40 minutes’ long | Continued emphasis on the importance of cervical cancer screening, how to prevent it and how to perform the screening | In this session, a gynecologist teaches the importance of cervical cancer screening, how to prevent it, and how to perform the cervical cancer screening through lectures, active collaborative discussion, and practical demonstration. |
| 9^rd^ week/60 minutes’ long | Providing useful strategies to remove the barriers to screening for cervical cancer to strengthen the enabling factors | In this session, participatory discussion and brainstorming are used to enlist the perceived barriers. Then, suggestions are made to remove these barriers through brainstorming. |
| 10^th^ week/60 minutes’ long | Review of all educational materials by gynecologists | In this session, the gynecologist briefly reviews all the previous materials, emphasizing the importance of the cervical cancer screening and early diagnosis of the cancer. |
| **Interpersonal level** | | |
| 1^st^ week/40 minutes’ long | Overview of cervical cancer, importance of cervical cancer screening test | In this session, those living with or closely related to the participating women took part to learn about cervical cancer and the significance of the screening test. The teaching methods included lectures, active participatory discussion, and questions and answers |
| 2nd week/40 minutes’ long | Knowing how to prevent cervical cancer in women | In this session, the participants are introduced to the ways of preventing cervical cancer in women. This content is instructed through lectures, active participatory discussion, brainstorming, and peer education. |
| 3^rd^ week/40 minutes’ long | Supporting women to take the test | In this session, the trainer encourages women to conduct the screening test. The teaching methods include lectures, brainstorming and question and answer about the significance of the screening test, and the need for the family and friends’ support of this healthy behavior. |
| 4^th^ week/40 minutes’ long | Useful strategies to remove restrictions and encourage women to take the test | In this session, the participants get to know the ways to reduce the limitations and barriers to cervical cancer screening by the trainer through the brainstorming method. |
